# Supplementary material for: Novel Driver Strength Index highlights important cancer genes in TCGA PanCanAtlas patients
Source: PeerJ. 2022 Aug 11;10:e13860. doi: 10.7717/peerj.13860 (PMC9375969; doi:10.7717/peerj.13860)
Supplement: Supplemental Information 2 [file peerj-10-13860-s002.zip › COHORTS/genes plots/detailed/2021_8_16_14_9_distribution_events_detailed_4.pdf]

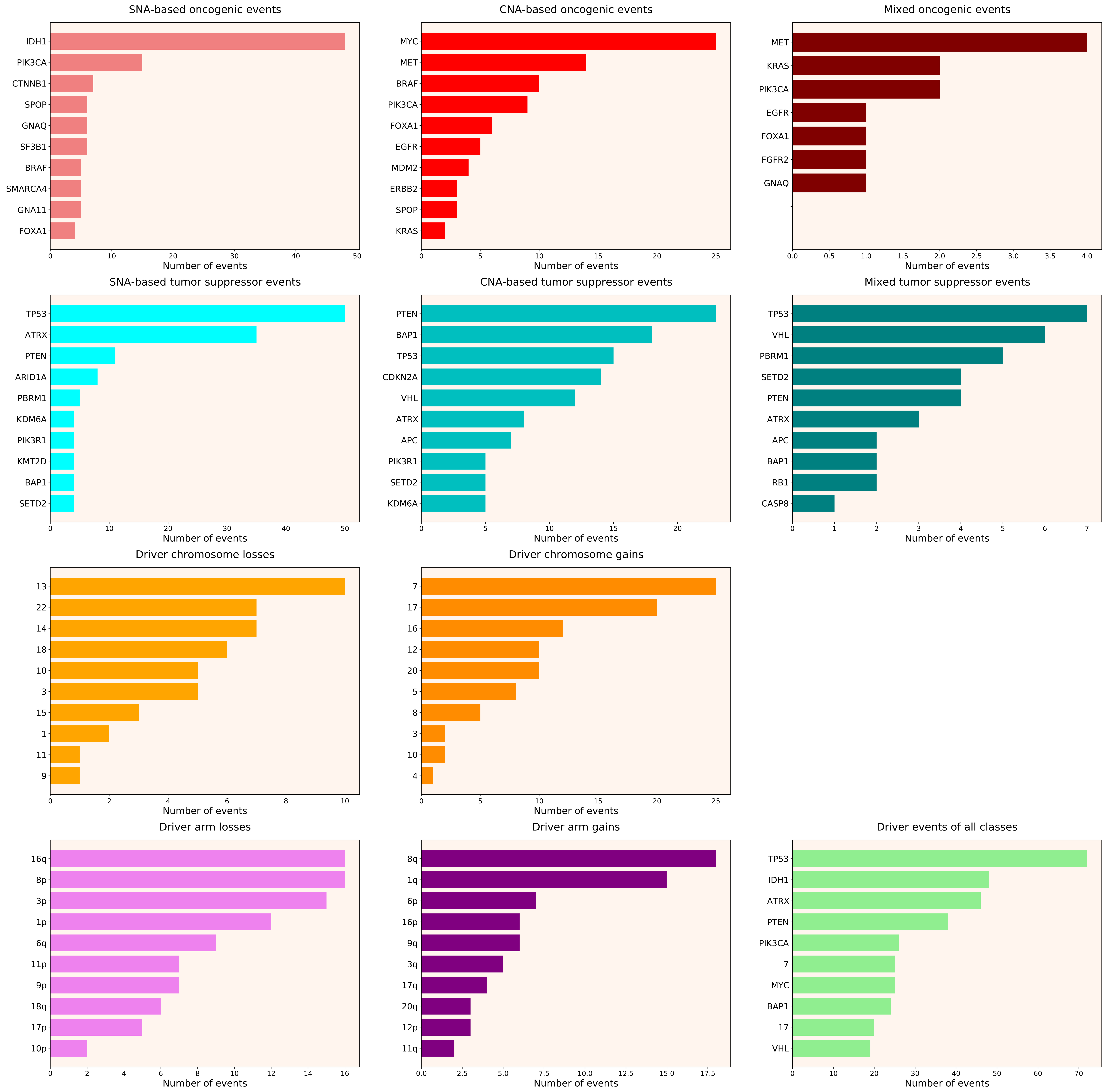

SNA-based oncogenic events
IDH1
PIK3CA
CTNNB1
SPOP
GNAQ
SF3B1
BRAF
SMARCA4
GNA11
FOXA1
Number of events
CNA-based oncogenic events
MYC
MET
BRAF
PIK3CA
FOXA1
EGFR
MDM2
ERBB2
SPOP
KRAS
Number of events
Mixed oncogenic events
MET
KRAS
PIK3CA
EGFR
FOXA1
FGFR2
GNAQ
Number of events
SNA-based tumor suppressor events
TP53
ATRX
PTEN
ARID1A
PBRM1
KDM6A
PIK3R1
KMT2D
BAP1
SETD2
Number of events
CNA-based tumor suppressor events
PTEN
BAP1
TP53
CDKN2A
VHL
ATRX
APC
PIK3R1
SETD2
KDM6A
Number of events
Mixed tumor suppressor events
TP53
VHL
PBRM1
SETD2
PTEN
ATRX
APC
BAP1
RB1
CASP8
Number of events
Driver chromosome losses
13
22
14
18
10
3
15
1
11
9
Number of events
Driver chromosome gains
7
17
16
12
20
5
8
3
10
4
Number of events
Driver arm losses
16q
8p
3p
1p
6q
11p
9p
18q
17p
10p
Number of events
Driver arm gains
8q
1q
6p
16p
9q
3q
17q
20q
12p
11q
Number of events
Driver events of all classes
TP53
IDH1
ATRX
PTEN
PIK3CA
7
MYC
BAP1
17
VHL
Number of events
